# Supplementary material for: Soil Fungal Composition Drives Ecosystem Multifunctionality after Long-Term Field Nitrogen and Phosphorus Addition in Alpine Meadows on the Tibetan Plateau
Source: Plants (Basel). 2022 Oct 28;11(21):2893. doi: 10.3390/plants11212893 (PMC9656404; doi:10.3390/plants11212893)
Supplement: Supplementary file 1 [file plants-11-02893-s001.zip › plants-1946970-supplementary.pdf]

Supplementary materials

Table S1. Linear correlations between ecosystem parameters and abiotic factors, including aboveground biomass (AGB); belowground biomass (BGB); roots organic carbon (ROC); microbial biomass carbon (MBC); soil organic carbon (SOC); cumulative carbon mineralization (CCM); microbial biomass nitrogen (MBN); soil nitrate nitrogen (NO_3^-–N); roots total nitrogen (RTN); soil ammonia nitrogen (NH_4^+–N); soil available phosphorus (SAP); ecosystem multifunctionality (EMF); soil acidity and alkalinity (pH); soil bulk density (SBD); relative light conditions (RLC); soil water content (SWC).

Abiotic factors	Ecosystem parameters	Linear correlations
SBD	AGB	Y=764.34-279.91X (R^2=0.02, p > 0.05)
	BGB	Y=3225.17-815.02X (R^2=0.01, p > 0.05)
	ROC	Y=383.70-20.62X (R^2=0.01, p > 0.05)
	MBC	Y=637.51-28.25X (R^2=0.05, p > 0.05)
	SOC	Y=40.85-4.27X (R^2=0.008, p > 0.05)
	CCM	Y=411-87.82X (R^2=0.04, p > 0.05)
	RTN	Y=7.63+2.09X (R^2=0.03, p > 0.05)
	**MBN**	**Y=28.73+76.33X (R^2=0.25, p < 0.05)**
	NO_3^-–N	Y=33.64-8.62X (R^2=0.05, p > 0.05)
	NH_4^+–N	Y=2.31+0.77X (R^2=0.05, p > 0.05)
	SAP	Y=-29.68+43.47X (R^2=0.11, p > 0.05)
	EMF	Y=0.35+0.08X (R^2=0.02, p > 0.05)
SWC	AGB	Y=156.95+576.6X (R^2=0.05, p > 0.05)
	BGB	Y=1072.61+2800.32X (R^2=0.07, p > 0.05)
	ROC	Y=307.87+133.19X (R^2=0.10, p > 0.05)
	MBC	Y=521.24+218.70X (R^2=0.01, p > 0.05)
	SOC	Y=32.41+6.39X (R^2=0.02, p > 0.05)
	CCM	Y=198.57+244.76X (R^2=0.05, p > 0.05)
	RTN	Y=10.66+0.09X (R^2=0.05, p > 0.05)
	MBN	Y=173.50-96.31X (R^2=0.08, p > 0.05)
	NO_3^-–N	Y=10.17+31.70X (R^2=0.1, p > 0.05)
	NH_4^+–N	Y=4.38-2.75X (R^2=0.11, p > 0.05)
	SAP	Y=47.70-40.08X (R^2=0.02, p > 0.05)
	EMF	Y=0.36+0.29X (R^2=0.05, p > 0.05)
pH	**AGB**	**Y=2544.06-312.78X (R^2=0.22, p < 0.05)**
	BGB	Y=8806.58-967.78X (R^2=0.12, p > 0.05)
	ROC	Y=30.83+46.09X (R^2=0.17, p > 0.05)
	**MBC**	**Y=-1817.04+344.72X (R^2=0.29, p < 0.01)**
	SOC	Y=45.88-1.61X (R^2=0.02, p > 0.05)
	**CCM**	**Y=-816.79+157.02X (R^2=0.23, p < 0.05)**
	**RTN**	**Y=43.02-4.62X (R^2=0.43, p < 0.01)**
	MBN	Y=130.21+1.47X (R^2=0.05, p > 0.05)
	NO_3^-–N	Y=46.92-3.69X (R^2=0.02, p > 0.05)
	NH_4^+–N	Y=1.77+0.24X (R^2=0.01, p > 0.05)
	SAP	Y=129.47-13.64X (R^2=0.03, p > 0.05)
	EMF	Y=0.2+0.04X (R^2=0.01, p > 0.05)
RLC	**AGB**	**Y=531.70-809.12X (R^2=0.55, p < 0.001)**
	**BGB**	**Y=2492.19-2102.05X (R^2=0.23, p < 0.05)**

	ROC	Y=344.08+42.95X (R^2=0.06, $p > 0.05$)
	**MBC**	**Y=475.22+551.84X (R^2=0.33, $p < 0.01$)**
	**SOC**	**Y=36.42-8.29X (R^2=0.21, $p < 0.05$)**
	**CCM**	**Y=233.44+223.42X (R^2=0.22, $p < 0.05$)**
	**RTN**	**Y=12.21-6.89X (R^2=0.40, $p < 0.01$)**
	MBN	Y=137.33+14.57X (R^2=0.01, $p > 0.05$)
	**NO_3^-–N**	**Y=25.39-19.94X (R^2=0.23, $p < 0.05$)**
	NH_4^+–N	Y=3.16+1.29X (R^2=0.14, $p > 0.05$)
	SAP	Y=32.40+7.16X (R^2=0.002, $p > 0.05$)
	EMF	Y=0.47-0.02X (R^2=0.05, $p > 0.05$)

Table S2. Linear correlations between species richness and abiotic factors, including soil acidity and alkalinity (pH); soil bulk density (SBD); relative light conditions (RLC); soil water content (SWC).

Abiotic factors	Species richness	Linear correlations
SBD	Plant	Y=21.26-1.41X (R^2=0.05, $p > 0.05$)
	Bacterial	Y=2208.71+394.54X (R^2=0.04, $p > 0.05$)
	Fungal	Y=1628.51+145.92X (R^2=0.03, $p > 0.05$)
SWC	Plant	Y=22.11-8.49X (R^2=0.05, $p > 0.05$)
	Bacterial	Y=2981.59-569.66X (R^2=0.03, $p > 0.05$)
	Fungal	Y=1803.65+112.57X (R^2=0.05, $p > 0.05$)
pH	Plant	**Y=-134.09+21.89X (R^2=0.54, $p < 0.001$)**
	Bacterial	Y=2296.67+69.97X (R^2=0.05, $p > 0.05$)
	Fungal	Y=3110.91-181.23X (R^2=0.05, $p > 0.05$)
RLC	Plant	**Y=10.58+39.35X (R^2=0.68, $p < 0.001$)**
	Bacterial	Y=2858.57-328.94X (R^2=0.06, $p > 0.05$)
	Fungal	Y=1886.69-203.04X (R^2=0.05, $p > 0.05$)

Table S3. Linear correlations between ecosystem parameters and species richness, including aboveground biomass (AGB); belowground biomass (BGB); roots organic carbon (ROC); microbial biomass carbon (MBC); soil organic carbon (SOC); cumulative carbon mineralization (CCM); microbial biomass nitrogen (MBN); soil nitrate nitrogen (NO_3^-–N); roots total nitrogen (RTN); soil ammonia nitrogen (NH_4^+–N); soil available phosphorus (SAP); ecosystem multifunctionality (EMF).

Species richness	Ecosystem parameters	Linear correlations
Plant	**AGB**	**Y=674.46-16.67X (R^2=0.55, $p < 0.001$)**
	BGB	Y=2715.39-35.61X (R^2=0.15, $p > 0.05$)
	ROC	Y=338.67+0.77X (R^2=0.04, $p > 0.05$)
	MBC	Y=440.05+8.13X (R^2=0.16, $p > 0.05$)
	SOC	Y=37.25-0.14X (R^2=0.13, $p > 0.05$)
	CCM	Y=224.31+3.03X (R^2=0.09, $p > 0.05$)
	**RTN**	**Y=13.87-0.17X (R^2=0.52, $p < 0.001$)**
	MBN	Y=143.48-0.15X (R^2=0.05, $p > 0.05$)
	**NO_3^-–N**	**Y=29.98-0.47X (R^2=0.28, $p < 0.05$)**
	**NH_4^+–N**	**Y=2.74+0.04X (R^2=0.25, $p < 0.05$)**
	SAP	Y=-44.94-0.57X (R^2=0.04, $p > 0.05$)
	EMF	Y=0.53-0.004X (R^2=0.09, $p > 0.05$)

Bacterial	AGB	Y=-219.13+0.21X (R^2=0.07, $p > 0.05$)
	BGB	Y=-1027.99+1.09X (R^2=0.12, $p > 0.05$)
	ROC	Y=355.97-0.0008X (R^2=0.05, $p > 0.05$)
	MBC	Y=624.11-0.01X (R^2=0.05, $p > 0.05$)
	SOC	Y=25.09+0.003X (R^2=0.07, $p > 0.05$)
	CCM	Y=417.34-0.05X (R^2=0.02, $p > 0.05$)
	RTN	Y=17.09-0.002X (R^2=0.08, $p > 0.05$)
	MBN	Y=137.21+0.001X (R^2=0.05, $p > 0.05$)
	NO_3^-–N	Y=1.47+0.007X (R^2=0.05, $p > 0.05$)
	NH_4^+–N	Y=6.22-0.0009X (R^2=0.15, $p > 0.05$)
	SAP	Y=91.09-0.02X (R^2=0.04, $p > 0.05$)
	EMF	Y=0.59+0.0004X (R^2=0.01, $p > 0.05$)
Fungal	AGB	Y=72.63+0.15X (R^2=0.02, $p > 0.05$)
	BGB	Y=652.41+0.75X (R^2=0.02, $p > 0.05$)
	ROC	Y=369.73-0.008X (R^2=0.05, $p > 0.05$)
	MBC	Y=560.63+0.02X (R^2=0.05, $p > 0.01$)
	SOC	Y=45.04-0.005X (R^2=0.08, $p > 0.05$)
	CCM	Y=306.48-0.01X (R^2=0.05, $p >0.05$)
	RTN	Y=9.35+0.0007X (R^2=0.05, $p > 0.01$)
	MBN	Y=128.31+0.006X (R^2=0.05, $p > 0.05$)
	NO_3^-–N	Y=34.64-0.007X (R^2=0.03, $p > 0.05$)
	NH_4^+–N	Y=3.92-0.0002X (R^2=0.05, $p > 0.05$)
	SAP	Y=-30.55+0.04X (R^2=0.05, $p > 0.05$)
	EMF	Y=0.42+0.00002X (R^2=0.01, $p > 0.05$)

Table S4. Mantel′r and Mantel′p, including aboveground biomass (AGB); belowground biomass (BGB); roots organic carbon (ROC); microbial biomass carbon (MBC); soil organic carbon (SOC); cumulative carbon mineralization (CCM); microbial biomass nitrogen (MBN); soil nitrate nitrogen (NO_3^-–N); roots total nitrogen (RTN); soil ammonia nitrogen (NH_4^+–N); soil available phosphorus (SAP); ecosystem multifunctionality (EMF); soil acidity and alkalinity (pH); soil bulk density (SBD); relative light conditions (RLC); soil water content (SWC).

	**Abiotic factors and ecosystem parameters**	**Mantel′r**	**Mantel′*p***
Bacterial composition	SBD	0.09	0.21
	SWC	-0.05	0.61
	pH	-0.07	0.71
	RLC	0.03	0.42
	AGB	0.11	0.09
	**BGB**	**0.36**	**0.002**
	ROC	-0.005	0.52
	**MBC**	**0.41**	**0.001**
	**SOC**	**0.30**	**0.003**
	CCM	0.02	0.41
	RTN	-0.03	0.56
	**MBN**	**0.25**	**0.02**
	**NO_3^-–N**	**0.25**	**0.004**
	NH_4^+–N	0.09	0.24
	**SAP**	**0.44**	**0.001**
	EMF	-0.14	0.93

Fungal composition	SBD	-0.04	0.59
	SWC	0.05	0.32
	pH	0.05	0.34
	**RLC**	**0.23**	**0.01**
	**AGB**	**0.14**	**0.05**
	BGB	0.17	0.06
	**ROC**	**0.22**	**0.02**
	**MBC**	**0.22**	**0.02**
	SOC	0.15	0.07
	CCM	0.09	0.18
	RTN	0.02	0.41
	**MBN**	**0.02**	**0.007**
	**NO_3^-–N**	**0.29**	**0.002**
	**NH_4^+–N**	**0.34**	**0.002**
	**SAP**	**0.5**	**0.001**
	EMF	-0.02	0.54
Plant composition	SBD	0.04	0.40
	SWC	-0.19	0.92
	pH	0.07	0.30
	**PLC**	**0.20**	**0.04**
	**AGB**	**0.20**	**0.01**
	BGB	-0.03	0.56
	ROC	0.004	0.46
	**MBC**	**0.28**	**0.015**
	SOC	-0.02	0.55
	CCM	0.06	0.30
	RTN	0.16	0.13
	MBN	0.08	0.23
	NO_3^-–N	0.11	0.11
	NH_4^+–N	-0.07	0.69
	**SAP**	**0.22**	**0.01**
	EMF	-0.07	0.73

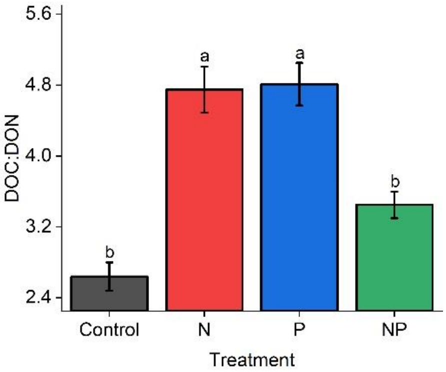

Figure S1. Effects of N and P addition on soil DOC: DON (M ± SE, n=5). Different letters indicate that the same index has significant differences among different treatments ($p<0.05$). Abbreviations: Control, the control without any nutrient addition; N, N-alone addition; P, P-alone addition; NP, N and P together addition; DOC, dissolved organic carbon; DON, dissolved organic nitrogen.

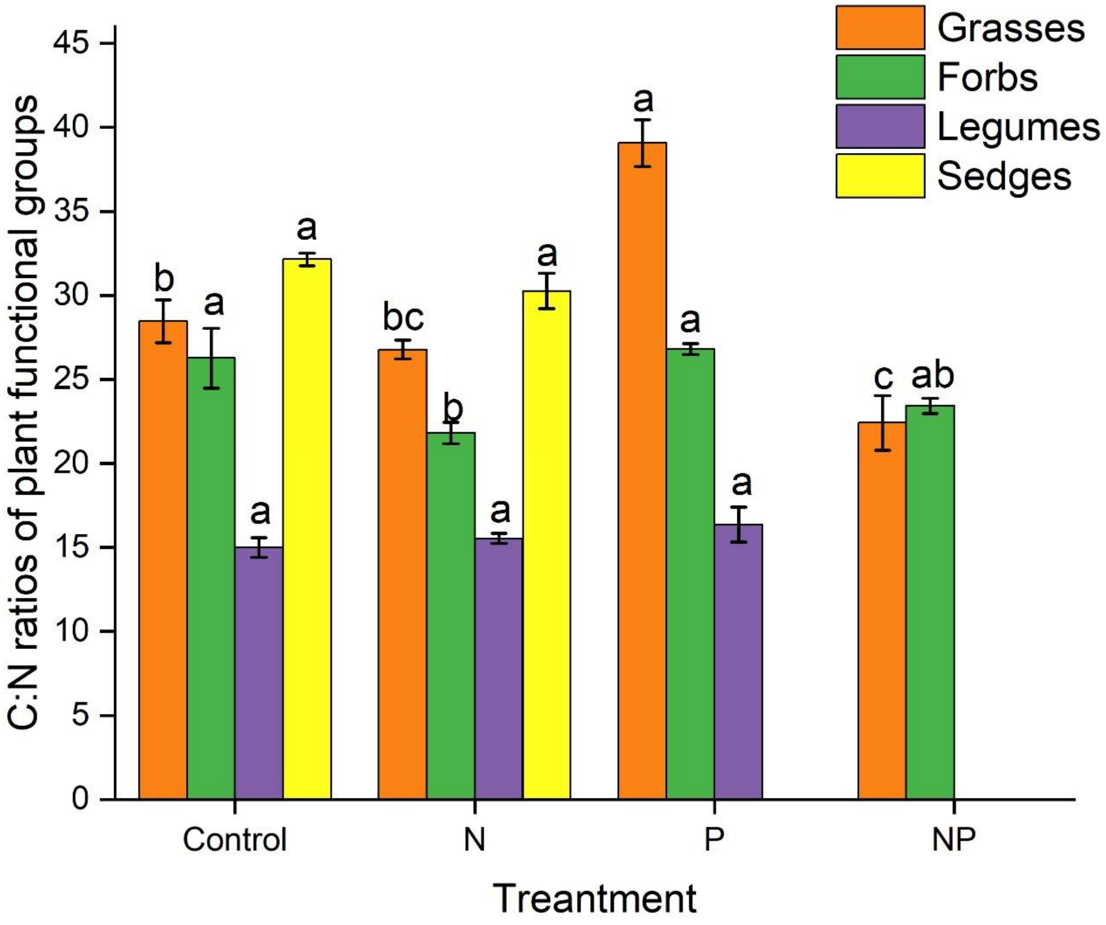

Figure S2. Effects of N and P addition on C: N ratios of plant functional groups (M ± SE, n=5). Different letters indicate that the same index has significant differences among different treatments ($p<0.05$). Abbreviations: Control, the control without any nutrient addition; N, N-alone addition; P, P-alone addition; NP, N and P together addition.

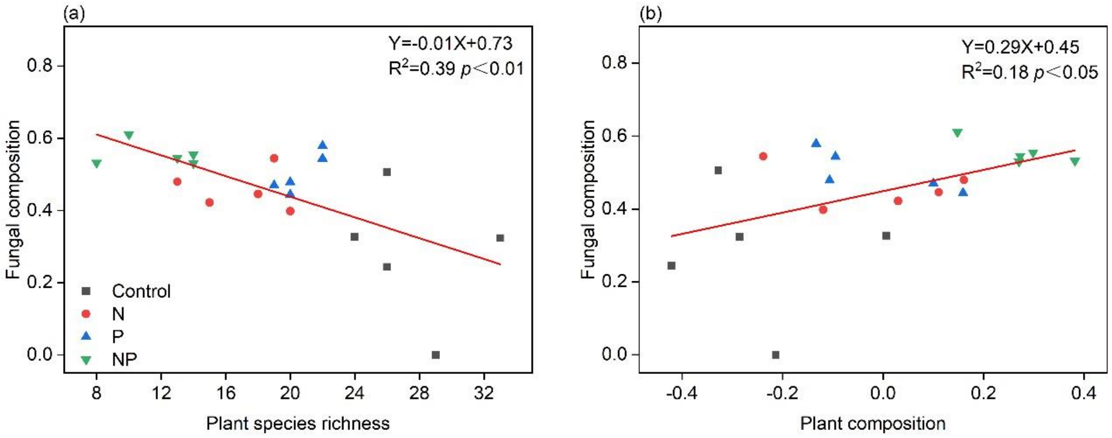

Figure S3. Linear correlations between fungal composition and plant diversity, including (a) fungal composition and plant species richness; (b) fungal composition and plant composition. Abbreviations: Control, the control without any nutrient addition; N, N-alone addition; P, P-alone addition; NP, N and P together addition.

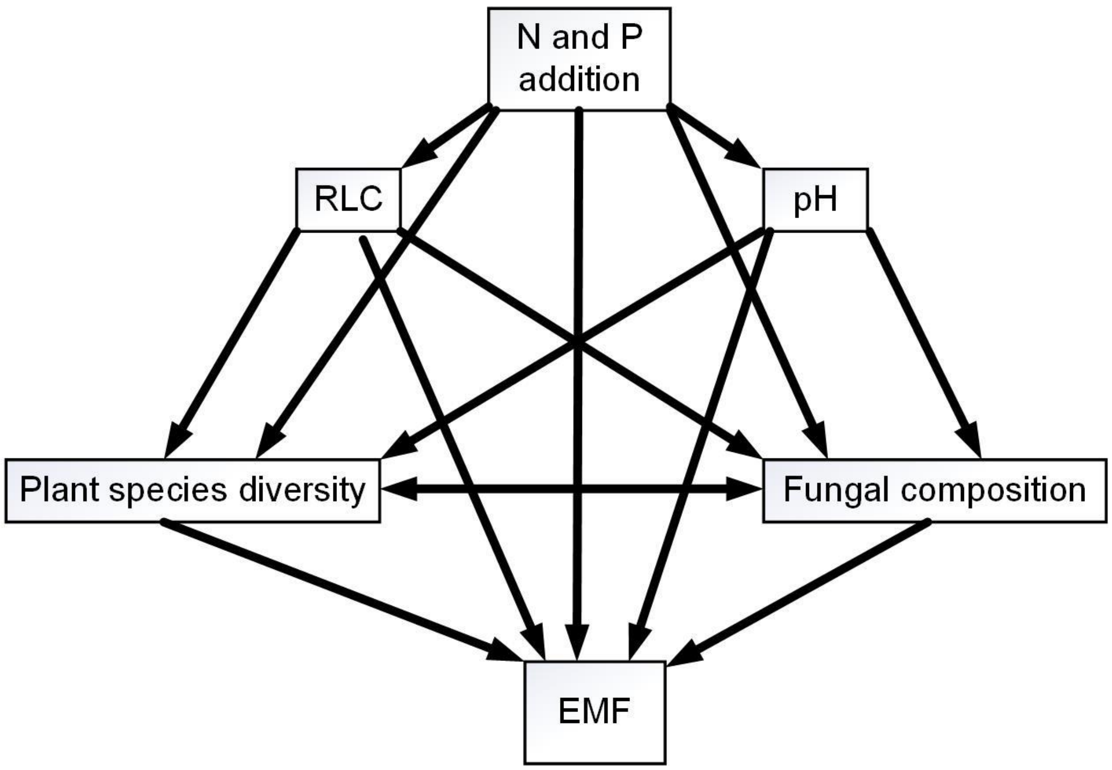

Figure S4. The priori model of structural equation modelling.
